# Insights into the Correlation between Residual Stresses, Phase Transformation, and Wettability of Femtosecond Laser-Irradiated Ductile Iron

**DOI:** 10.3390/nano12081271

**Published:** 2022-04-08

**Authors:** Dhiraj Kumar, Gerhard Liedl, Andreas Otto, Werner Artner

**Affiliations:** 1 Institute of Production Engineering and Photonic Technologies, TU Wien, 1030 Vienna, Austria; gerhard.liedl@tuwien.ac.at (G.L.); otto@ift.at (A.O.); 2 X-ray Center, TU Wien, 1060 Vienna, Austria; werner.artner@tuwien.ac.at

**Keywords:** femtosecond laser, wettability, phase change, residual stresses, EBSD, TEM

## Abstract

Despite numerous studies on the wettability behavior of ductile iron after ultrafast laser structuring, the correlation between the phase change due to the interaction with an intense pulse and wettability is not yet well understood. In the present work, phase transformations of ductile iron substrates after femtosecond laser irradiation are investigated and correlated with the wettability behavior. Laser parameters such as fluence (F), cumulative fluence (CH), number of pulses (N), and scan speed were varied to produce hierarchical structures with different morphologies and phase concentrations. Our outcomes indicated that substrates with higher concentrations of austenite in the absence of hierarchical structures have a superhydrophilic nature despite being stored in an ambient atmosphere for several days and the application of a vacuum process. In addition, we measured the concomitant residual stresses after laser irradiation using the X-ray diffraction (XRD) method and established a relationship with the doses of CH and induced micro/nanostructures. Transmission electron microscopy (TEM) revealed that laser-structured surfaces are covered with oxides; moreover, phase transformation occurs at the near-subsurface layer.

## 1. Introduction

Physical properties and surface characteristic modifications of metals after femtosecond (fs) laser irradiation have been investigated extensively in the past [1,2]. The innovative stimulus includes wetting, optical, and tribological properties [3,4]. These laser-functionalized surfaces have a wide range of applications that can be employed industrially. The intense pulse of the fs laser enables high-temperature and high-pressure shockwave processing of materials at exceptionally high isochoric heating (10^15^–10^16^ K/s) and cooling rates (10^12^ K/s) [5] that are accompanied by extreme sub-ablative subsurface melting [6]. However, such a process may cause chemical exclusion, yielding more volatile components in the surface concentration; consequently, a non-equilibrium phase transformation may induce residual stresses, which has been demonstrated in semiconductor materials [7]. Techniques based on picosecond timescale imaging and acoustic strain could be used to estimate the thermal properties and strain propagation profile of the thin film and nanostructure [8,9]. Several studies have shown improvement in the mechanical and physical properties of metal substrates using the laser shock peening (LSP) technique. LSP has not only altered the near-surface microstructure but also imparted compressive residual stresses; subsequently, it improves corrosion resistance and fatigue strength [10,11]. Nonetheless, based on our search, there is no evidence available of the correlation between heat accumulation, compressive residual stresses, and laser-induced hierarchical structures.

Moreover, in pure iron and silicon, it has been noticed that high-pressure metastable phases are formed when they are irradiated in the air with the fs laser [12]. Usually, phases have different free surface energies than their parent materials, which affect the surface wettability depending upon the percentage area or concentration of phases [13]. However, the influence of hierarchical structures and phase transformation in ductile iron’s near-surface layer on the wettability has not been investigated. In the present work, we intended to fill this gap for the ductile iron that has been widely used in automobiles, piping, and other components in water industries.

## 2. Materials and Methods

A Ti:sapphire femtosecond laser with an average power of 800 mW, operated at a 1000 Hz repetition rate with 30 fs pulse duration and 800 nm center wavelength (λ) was used to produce hierarchical micro/nanostructuring in the samples. Line scanning was performed with a linearly polarized beam having a smallest spot size of 35 ± 5 µm. A constant hatch distance of 40 µm was used to produce structures at varying CH (from 25 to 1591 J/cm^2^, achieved after changing scan speed from 0.2 mm/s to 1.0 mm/s and laser power from 50 mW to 250 mW). Further, treated samples were sonicated for 30 min in ethanol to clean the attached residues. Static contact angle (SCA) measurement was carried out using the sessile drop technique with a droplet (deionized water) of 2 μL. A Malvern Panalytical B.V. Empyrean diffractometer was used to perform stress measurements and phase analysis. A GaliPIX3D detector was used for detection of the diffracted beam. The detector to sample distance for this instrument is 240 mm. The XRD diagrams were evaluated using the Malvern Panalytical HighScore Plus v4.6a program suite. Electron backscatter diffraction (EBSD) observation was made on lamella taken from the treated and untreated samples using the focus ion beam (FIB) technique. Further analysis was conducted to investigate the phases and texture intensity of treated parts, along with grain size and orientation.

## 3. Results and Discussions

### 3.1. Residual Stress and Micrograph Analysis

**Figure 1:**
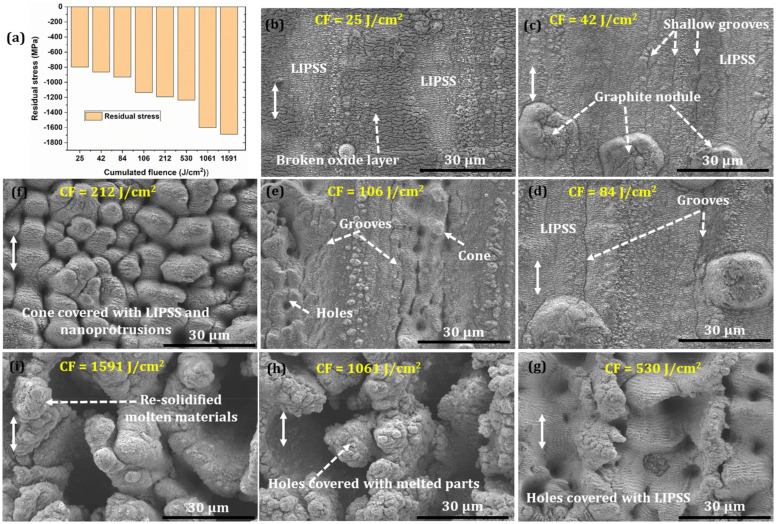
(**a**) Deviation of residual stresses at various CH and (**b**–**i**) corresponding microstructures. The white double head arrows denote the direction of polarization.

Due to the intense fs laser pulses, high-magnitude shock pressure developed in the materials responsible for the plastic deformation; as a result, compressive residual stress was generated in the material up to several micrometer depths [14]. However, in the present investigation, measurement depth was kept constant at 5 μm. Figure 1 demonstrates the compressive residual stresses deviation of cumulated fluence together with produced microstructures. It can be seen that compressive residual stress is increasing with an increase in CH which is attributed to more plastic deformation due to the shockwave generated by the fs laser at higher CH (see Figure 1a). Figure 1b–i shows the corresponding micrograph obtained after irradiating the ductile iron at the chosen cumulative fluence. At a lower CH (25 J/cm^2^, F = 0.1 J/cm^2^, N = 250), the formation of nanograting called laser-induced periodic surface structures (LIPSS) is observed, which are oriented perpendicular to the beam polarization (see Figure 1b, marked by the double-head arrow). In addition, it has also been seen that the nanograting structures are surrounded by brittle oxide layers, which has been confirmed by the EDAX spectra (see Figure S1).

Previous studies demonstrate the formation of LIPSS on these metal surfaces at a fluence close to the ablation threshold [15]. However, with a further increase in the number of pulses, the ablation threshold of materials decreases due to the cumulative effect. Therefore, we assumed that ablation might happen and melted the materials, redistributed, and organized due to the developed shock pressures and plastic deformation. However, a detailed experiment and analysis of the metal’s surface are required with varying pulses. Figure 1c shows the formation of shallow grooves and graphite nodules apart from the LIPSS. As discussed above, such a cumulative effect occurs due to the accumulation of plastic deformation, consequentially from the developed thermal stresses when the sample was irradiated at the same fluence but with increased pulses (N = 416). Therefore, plastic deformation at a higher number of pulses could be a reason for the formation of shallow grooves. Moreover, graphite nodule presence is accredited to the rapid solidification of the molten materials, as discussed in [16]. The main constituents of graphite aggregates are the hexagonal graphite nanoplatelets with height and depth in nanometer and micrometer scales, respectively. During the solidification process, platelets thickening occurs due to the layer-by-layer graphene nucleation at the shelves of the graphite prism [17]. However, a detailed investigation of the mechanism associated with graphite nodule formation is not in the scope of the current manuscript.

Likewise, a similar micrograph with deeper grooves is noticed with a further increase in CH (see Figure 1d). At 106 J/cm^2^ (F = 0.63 J/cm^2^, N = 166), hole-like structures with deeper grooves and cones appear on the surface due to the accumulated heat, as shown in Figure 1e. However, at 212 J/cm^2^ (F = 0.50 J/cm^2^, N = 416), a combination of hole- and cone-like structures decorated with ripples and nanoprotrusions appeared (see Figure 1f). A similar phenomenon can be seen in Figure 1g, with a wider opening and solidified molten materials. Shockwave-induced plastic deformation could be the reason for such structures, as it also surges the compressive residual stress from 1192 MPa to 1237 MPa. A hole-like structure and resolidified molten material along the periphery without appearance of ripples are observed when irradiated at 1061 J/cm^2^ (F = 1.41 J/cm^2^, N = 750) and 1591 J/cm^2^ (F = 3.18 J/cm^2^, N = 1250) (Figure 1h,i). Laser ablation mechanisms such as spallation and phase explosion could be a reason as the pulses per shot are significantly higher [18], which may have augmented the heat accumulation and plastic deformation. It can be corroborated with a marginal increase in compressive residual stress, from 1600 MPa to 1690 MPa.

### 3.2. Phase Transformation of the Near-Surface Layer

**Figure 2:**
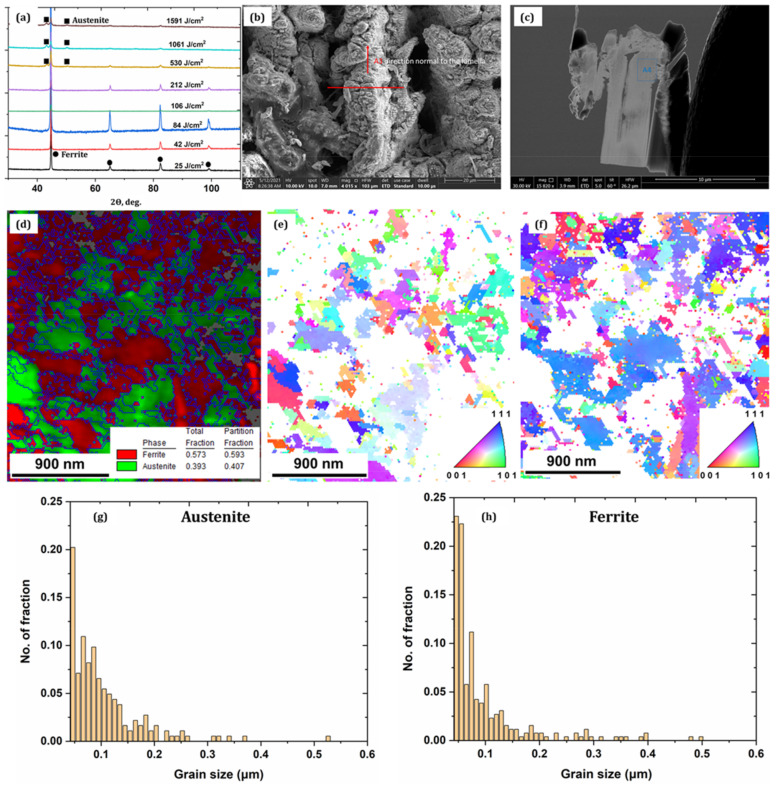
(**a**) XRD spectrum for the samples irradiated at several CH; (**b**) A sample morphology irradiated at 1561 J/cm^2^ used for the EBSD sample preparation; (**c**) cross-section lamella taken using FIB; (**d**) EBSD phase analysis that showed the presence of the austenite phase; (**e**,**f**) EBSD inverse pole map for the austenite and ferrite phases, respectively; (**g**,**h**) grain size distribution of the austenite and ferrite phases, respectively.

Figure 2a shows the XRD patterns of the ductile iron irradiated at various cumulative fluences. The surface illuminated from 25 to 212 J/cm^2^ demonstrates the ferritic phase of the non-irradiated sample surface. The rapid cooling at lower CH might be the one reason that restricts austenite formation, while with a further increase in cumulative fluence, the austenitic phase appears with varying concentrations. For example, at 530 J/cm^2^, the concentration of the austenite phase in the fine layer surface is ~19%; however, at higher CH between 1061 J/cm^2^ and 1561 J/cm^2^, the observed austenite concentrations are ~45% and ~49%, respectively. Figure 2b,c shows the micrograph of the sample and lamella taken for the EBSD analysis using the FIB technique from the sample irradiated at 1561 J/cm^2^. Figure 2d–f shows the phase analysis and inverse pole figure of the austenite and ferrite phases that indicate the presence of austenite with a phase volume of ~40% and a grain size ranging from 0.04 to 0.52 μm. The ferritic phase volume of ~60% with grain size ranging from 0.04 to 0.52 μm is noticed; moreover, the untreated ferritic grain size was in a range from 1.77 to 48.46 μm. The average grain sizes of austenite and ferrite are 0.21 μm and 0.25 μm, respectively (see Figure 2g,h). From the above observation, one can infer that as the number of pulses per shot increases, the accumulated heat restricts rapid cooling of the molten pool, therefore increasing the nucleation rate during the solidification and promoting the formation of fine-grain structure.

**Figure 3:**
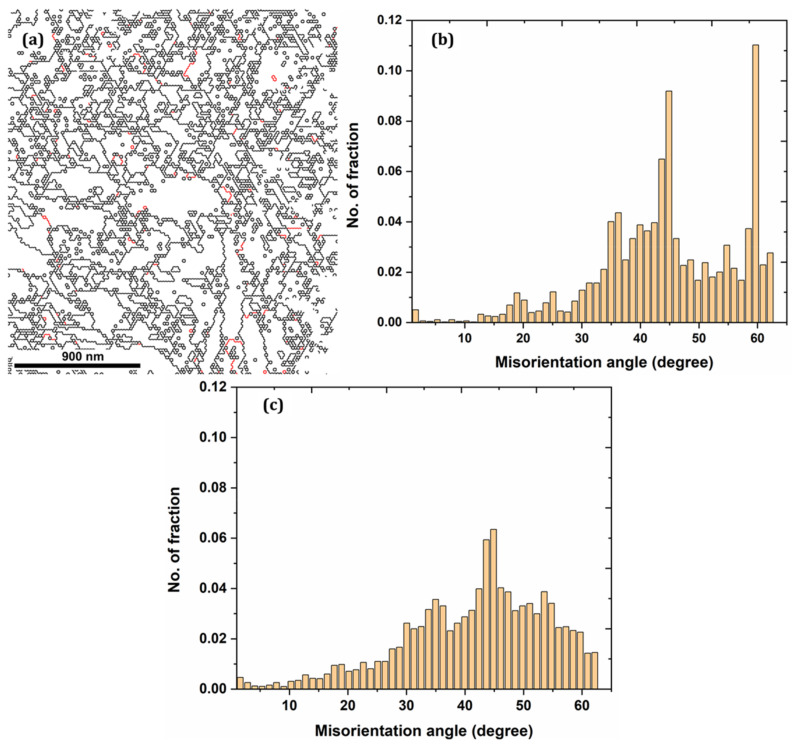
(**a**) High- and low-angle grain boundary distribution; (**b**,**c**) Misorientation angles for the austenite and ferrite phases, respectively.

**Figure 4:**
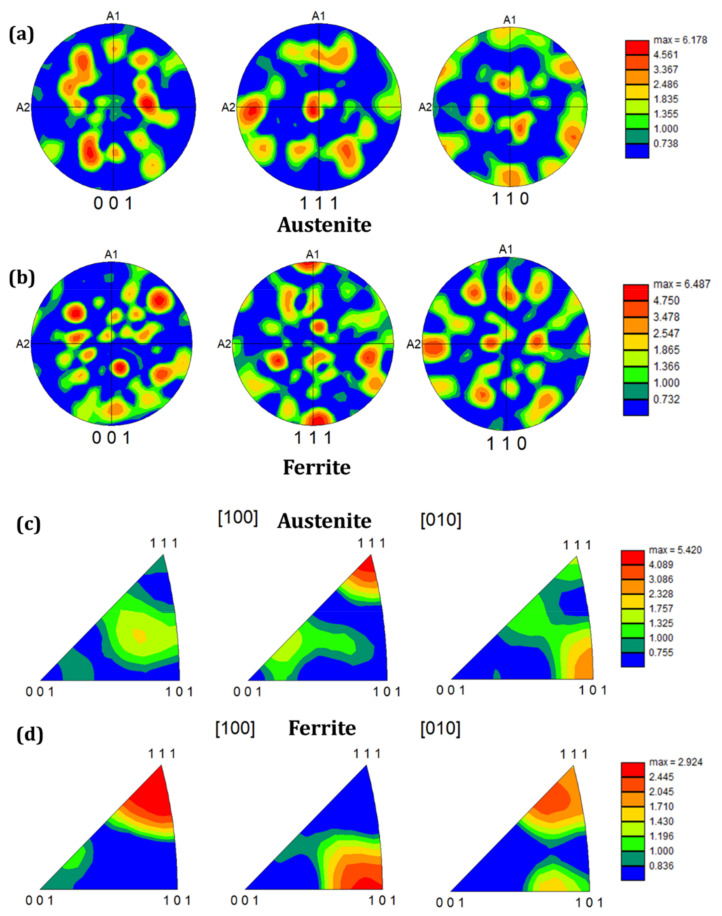
Pole figure and inverse pole figure (**a**,**c**) of the austenite phase; and (**b**,**d**) ferrite phase.

Figure 3a shows the size of the angle grain boundary distribution, in which the high-angle grain boundary (HAGB) (greater than 15°) is black, and the low-angle grain boundary (LAGB) (less than 15°) is red. The fraction of LAGB at the near-surface is more significant, especially at ~2–5° LAGB, which are considered sub-grain boundaries formed due to the dislocation rearrangement. Therefore, one can deduce that grain refinements have led to the formation of many sub-grain boundaries. Figure 3b,c presents the misorientation angle of the austenite and ferrite phases, respectively. Average austenite and ferrite phase values are 44.48° and 41.67°, respectively. It can be seen from Figure 4a,b that the maximum texture intensity close to the laser-irradiated area increases from 3.955 to 6.178 and 4.670 to 6.487 for austenite and ferrite, respectively, when compared to far away from the top surface (see Figure S2). This indicates that texture was enhanced at the near-surface layer; therefore, the density of the crystal grain in a specific direction increases, and the force between the crystal grain is enhanced. Moreover, the austenite phase has the texture in the (100) axis direction and texture strength in the (111) direction. However, the ferrite phase has apparent texture in the (001) axis direction and texture strength in a similar direction as the austenite phase (see Figure 4c,d).

### 3.3. TEM Characterization

**Figure 5:**
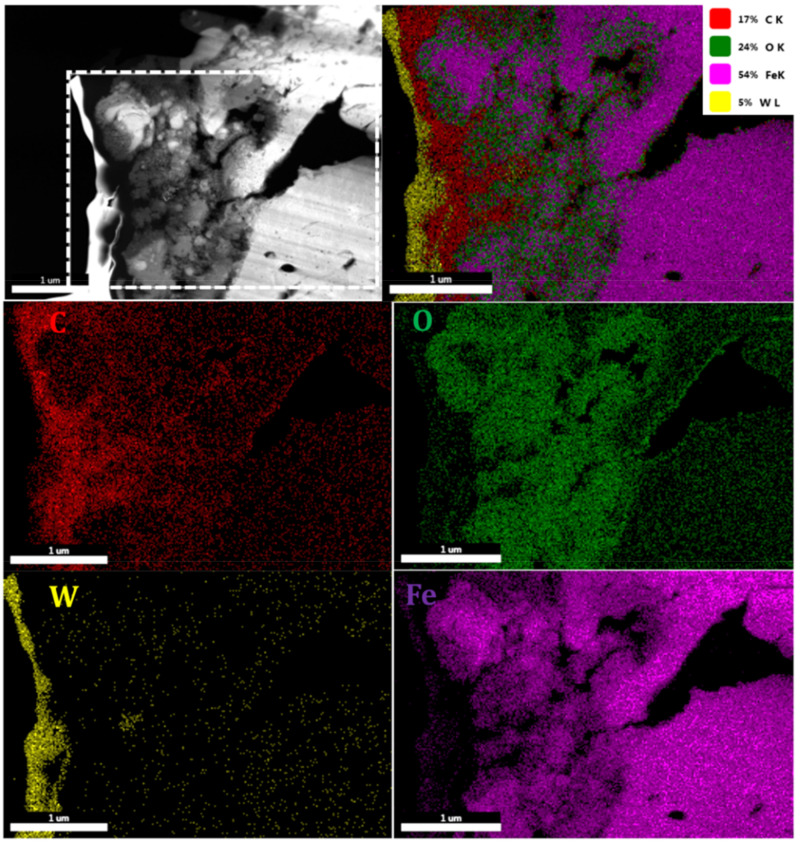
TEM-EDAX mapping of the laser-structured sample (F = 3.18 J/cm^2^; CH = 1561 J/cm^2^).

**Figure 6:**
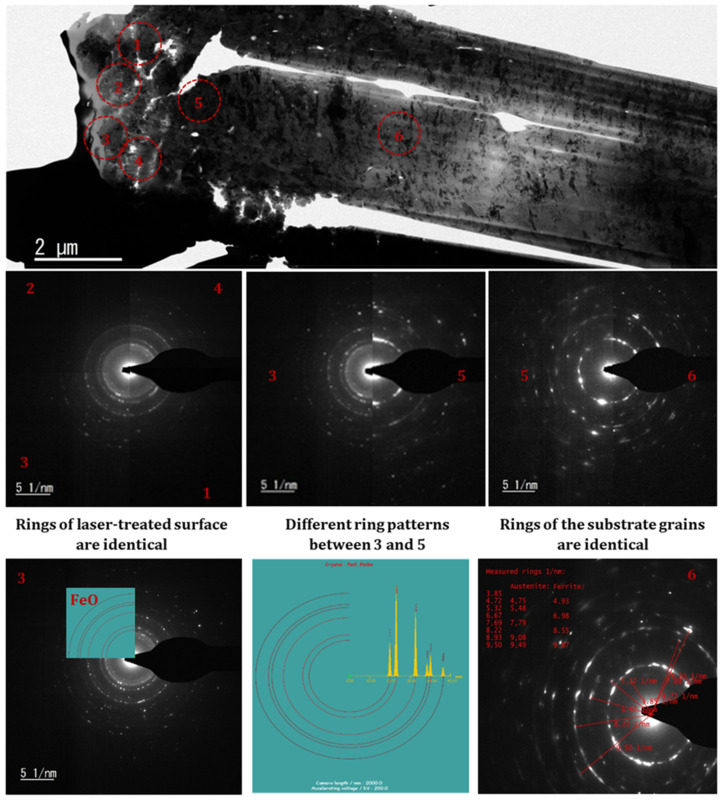
TEM analysis of the sample irradiated at CH = 1561 J/cm^2^.

TEM characterization of the cross-sectional sample obtained after FIB was completed and the results are shown in Figure 5 and Figure 6. Figure 5 represents the element mapping of the upper laser-structured surface which reveals 24% oxygen content and 17% carbon. Tungsten (W) is from the FIB protective layers, which cluster together and can be witnessed above the surface. Therefore, one can deduce that laser structuring in the ambient atmosphere leads to the formation of oxides over the surfaces.

Figure 6 shows the diffraction pattern of the sample taken at various locations. Diffraction patterns obtained at locations one, two, three, and four are identical, captured at the top layer of the irradiated surface that accords with FeO rings, as shown in the bottom of the figure (see bottom left and middle). On the other hand, the diffraction pattern of location five is not identical to the top layers, signifying different crystal structures near the surface layer. Moreover, locations five and six have similar ring patterns that show the crystal structure has been modified up to a certain depth from the top surfaces. In addition, the phase analysis of the substrate has been carried out based on the diffraction ring. The ferritic and austenitic phases are witnessed on the substrates as confirmed by the XRD and EBSD characterizations.

### 3.4. Wettability Analysis

**Figure 7:**
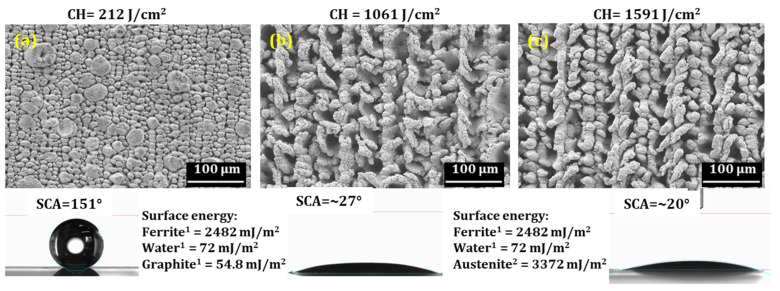
SCA comparison with structures and different phases obtained at various CH (**a**) 212 J/cm^2^, (**b**) 1061 J/cm^2^ and (**c**) 1591 J/cm^2^.

Figure 7 shows the micrograph of samples with their corresponding SCA irradiated at three different fluences. As discussed above, structures obtained at 212 J/cm^2^ do not show phase transformation; however, at higher CH, the austenite phase is observed. SCA measurement was completed after several days of exposure to the ambient atmosphere and after applying the vacuum process, as discussed in an earlier study [19]. It can be seen that the absence of a hierarchical structure, together with a higher concentration of austenitic phases leads to the hydrophilic nature of the surface. It is a well-known fact that surface energy plays a significant role in achieving any surface’s hydrophobic or superhydrophobic behavior. Therefore, a surface with higher surface energy could be readily wetted by a liquid with low surface energy. For example, the surface energy value of water is 72 mJ/m^2^ [20]; thus, a material with higher surface energy can be easily wetted and has an SCA lower than 90°.

Moreover, a material with lower surface energies than water will not adhere to the surface easily; as a result, it will have an SCA higher than 90°. In the present investigation, a graphite nodule has lower surface energy (54.8 mJ/m^2^ [21]) than water which could be a reason, together with the hierarchical structures that the surface showed superhydrophobic behavior, despite having a ferritic phase with higher surface energy (see Figure 7a). However, the graphite nodules disappeared at high CH (a detailed analysis on ablation was demonstrated in an earlier study [19]) and chaotic structures with austenite phases (concentrations of ~45% and ~49%, respectively, at 1061 J/cm^2^ and 1591 J/cm^2^) are observed that have higher surface energy leading to a lower SCA, as shown in Figure 7b,c. In addition, numerous studies have been conducted to correlate surface roughness with SCA [22,23,24]. It has been noticed that the SCA increases as the surface roughness increases [24]. Therefore, a surface roughness measurement of the samples irradiated at 212 J/cm^2^ and 1591 J/cm^2^ was carried out using an optical profilometer (see Figure S3). It can be seen that surface roughness increases with an increase in CH; however, the SCA doesn’t show any improvement. Therefore, one can deduce that phases with different surface energies without hierarchical structures significantly impact the SCA, regardless of higher surface roughness. However, further investigations are required to clearly understand the correlation between SCA and austenite phase concentration by producing a surface structure with austenitic phases.

## 4. Conclusions

Phase transformation and residual stress after femtosecond laser surface structuring of ductile iron have been investigated and correlated to its wettability behavior. Different structures have been generated at various CH levels that showed an increase in the magnitude of compressive residual stresses with increasing CH. Heat accumulation and plastic deformation were identified as influencing parameters for the varieties of surface structures. XRD analysis showed phase transformation from ferritic to austenitic at the near-surface layer at a sufficiently high CH. EBSD investigation revealed refined grains and grain boundaries at the sub-surface layer. Moreover, texture strength has also improved in the region close to the irradiated surface. Surfaces in the absence of hierarchical morphology with the austenite phase showed as superhydrophilic in nature even after several days of exposure to ambient conditions.

## Data Availability

Not applicable.

## Supplementary Materials

The following supporting information can be downloaded at: https://www.mdpi.com/article/10.3390/nano12081271/s1, Figure S1: (a) SEM micrograph with area of interest (marked in white dotted line), (b) obtained EDAX spectra, and (c) the corresponding elemental concentration; Figure S2: Texture intensity of the samples taken deeper from the laser structured surface (Area of interest marked in green); Figure S3: 3D-surface topography and the corresponding Sa values at 212 J/cm^2^ and 1591 J/cm^2^.

## References

[1] Sugioka K., Cheng Y. (2014). Ultrafast lasers—Reliable tools for advanced materials processing. Light Sci. Appl..

[2] Cubero Á., Martínez E., Angurel L.A., de la Fuente G.F., Navarro R., Legall H., Krüger J., Bonse J. (2020). Surface Superconductivity Changes of Niobium Sheets by Femtosecond Laser-Induced Periodic Nanostructures. Nanomaterials.

[3] Zaum C., Osterloh N., Darkins R., Duffy D.M., Morgenstern K. (2021). Real-space observation of surface structuring induced by ultra-fast-laser illumination far below the melting threshold. Sci. Rep..

[4] Vlahou M., Fraggelakis F., Manganas P., Tsibidis G.D., Ranella A., Stratakis E. (2022). Fabrication of Biomimetic 2D Nanostructures through Irradiation of Stainless Steel Surfaces with Double Femtosecond Pulses. Nanomaterials.

[5] Ionin A.A., Kudryashov S.I., Seleznev L.V., Sinitsyn D.V., Bunkin A.F., Lednev V.N., Pershin S.M. (2013). Thermal melting and ablation of silicon by femtosecond laser radiation. J. Exp. Theor. Phys..

[6] Ionin A.A., Kudryashov S.I., Seleznev L.V., Sinitsyn D.V. (2011). Generation and detection of superstrong shock waves during ablation of an aluminum surface by intense femtosecond laser pulses. J. Exp. Theor. Phys. Lett..

[7] Smith M.J., Sehr M., Franta B., Lin Y., Mazur E., Gradecak S. (2012). The origins of pressure-induced phase transformations during the surface texturing of silicon using femtosecond laser irradiation. J. Appl. Phys..

[8] Dehoux T., Wright O.B., LiVoti R. (2010). Picosecond time scale imaging of mechanical contacts. Ultrasonics.

[9] Tomoda M., Matsuda O., Wright O.B. (2006). Tomographic reconstruction of picosecond acoustic strain propagation. Appl. Phys. Lett..

[10] Liu D., Shi Y., Liu J., Wen L. (2019). Effect of laser shock peening on corrosion resistance of 316L stainless steel laser welded joint. Surf. Coat. Technol..

[11] Yang J.M., Her Y.C., Han N., Clauer A. (2001). Laser shock peening on fatigue behavior of 2024-T3 Al alloy with fastener holes and stopholes. Mater. Sci. Eng. A.

[12] Smith M.J., Lin Y.T., Sher M.J., Winkler M.T., Mazur E., Gradecak S. (2011). Pressure-induced phase transformations during femtosecond-laser doping of silicon. J. Appl. Phys..

[13] Kietzig A.M., Hatzikiriakos S.G., Englezos P. (2009). Patterned superhydrophobic metallic surfaces. Langmuir.

[14] Nakhoul A., Rudenko A., Sedao X., Peillon N., Colombier J.P., Maurice C., Blanc G., Borbély A., Faure N., Kermouche G. (2021). Energy feedthrough and microstructure evolution during direct laser peening of aluminum in femtosecond and picosecond regimes. J. Appl. Phys..

[15] Kumar D., Liedl G., Gururaja S. (2020). Formation of sub-wavelength laser induced periodic surface structure and wettability transformation of CFRP laminates using ultra-fast laser. Mater. Lett..

[16] Ghassemali E., Hernando J.C., Stefanescu D.M., Dioszegi A., Jarfors A.E.W., Dluhoš J., Petrenec M. (2019). Revisiting the graphite nodule in ductile iron. Scr. Mater..

[17] Azeem M.A., Bjerre M.K., Atwood R.C., Tiedje N., Lee P.D. (2018). Synchrotron quantification of graphite nodule evolution during the solidification of cast iron. Acta Mater..

[18] Zhidkov M.V., Vershinin T.N., Golosova O.A., Kudryashov S.I., Ionin A.A. (2020). Surface texturing of steel by femtosecond laser and accompanying structure/phase transformations. Opt. Laser Technol..

[19] Kumar D., Sauer M., Ching K.K., Kalss G., Santos A.C.V.D.d., Ramer G., Foelske A., Lendl B., Liedl G., Otto A. (2021). Wettability transition of femtosecond laser patterned nodular cast iron (NCI) substrate. Appl. Surf. Sci..

[20] Vargaftik N.B., Volkov B.N., Voljak L.D. (1983). International Tables of the Surface Tension of Water. J. Phys. Chem. Ref. Data.

[21] Kozbial A., Li Z., Conaway C., McGinley R., Dhingra S., Vahdat V., Zhou F., D’Urso B., Liu H., Li L. (2014). Study on the surface energy of graphene by contact angle measurements. Langmuir.

[22] Wang J., Wu Y., Cao Y., Li G., Liao Y. (2020). Influence of surface roughness on contact angle hysteresis and spreading work. Colloid Polym. Sci..

[23] Bell M.S., Shahraz A., Fichthorn K.A., Borhan A. (2015). Effects of hierarchical surface roughness on droplet contact angle. Langmuir.

[24] Behera S.K., Ajay K.P., Dogra N., Nosonovsky M., Rohatgi P. (2019). Effect of microstructure on contact angle and corrosion of ductile iron: Iron-graphite composite. Langmuir.

