# Supplementary material for: Insights into the Correlation between Residual Stresses, Phase Transformation, and Wettability of Femtosecond Laser-Irradiated Ductile Iron
Source: Nanomaterials (Basel). 2022 Apr 8;12(8):1271. doi: 10.3390/nano12081271 (PMC9028523; doi:10.3390/nano12081271)
Supplement: Supplementary file 1 [file nanomaterials-12-01271-s001.zip › nanomaterials-1632814-supplementary.pdf]

nanomaterials

Supplementary information

Insights into the Correlation between Residual Stresses, Phase Transformation, and Wettability of Femtosecond Laser-Irradiated Ductile Iron

Dhiraj Kumar [1,*], Gerhard Liedl [1], Andreas Otto [1] and Werner Artner [2]

1 Institute of Production Engineering and Photonic Technologies, TU Wien, Vienna 1030, Austria; gerhard.liedl@tuwien.ac.at (G.L.); otto@ift.at (A.O.)
2 X-ray Center, TU Wien, Vienna 1060, Austria; werner.artner@tuwien.ac.at
* Correspondence: dhiraj.kumar@tuwien.ac.at

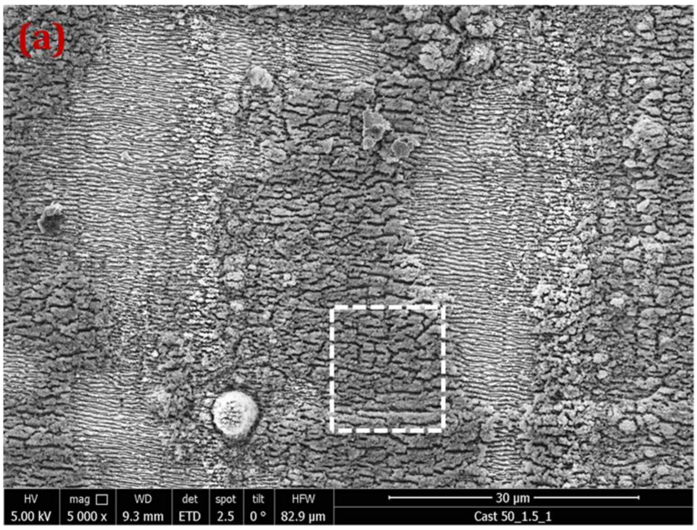

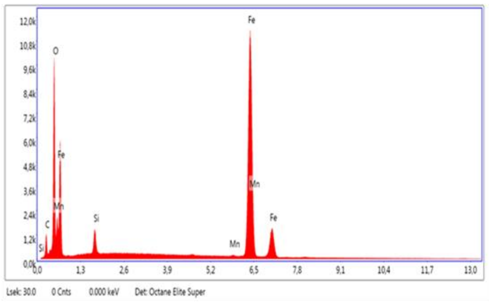

(c)

Element	Wt%	Atom%
C K	7.6	19.8
O K	19.1	37.6
SiK	1.9	2.1
MnK	0.3	0.2
FeK	71.2	40.2

Figure S1. (**a**) SEM micrograph with area of interest (marked in white dotted line), (**b**) obtained EDAX spectra, and (**c**) the corresponding elemental concentration.

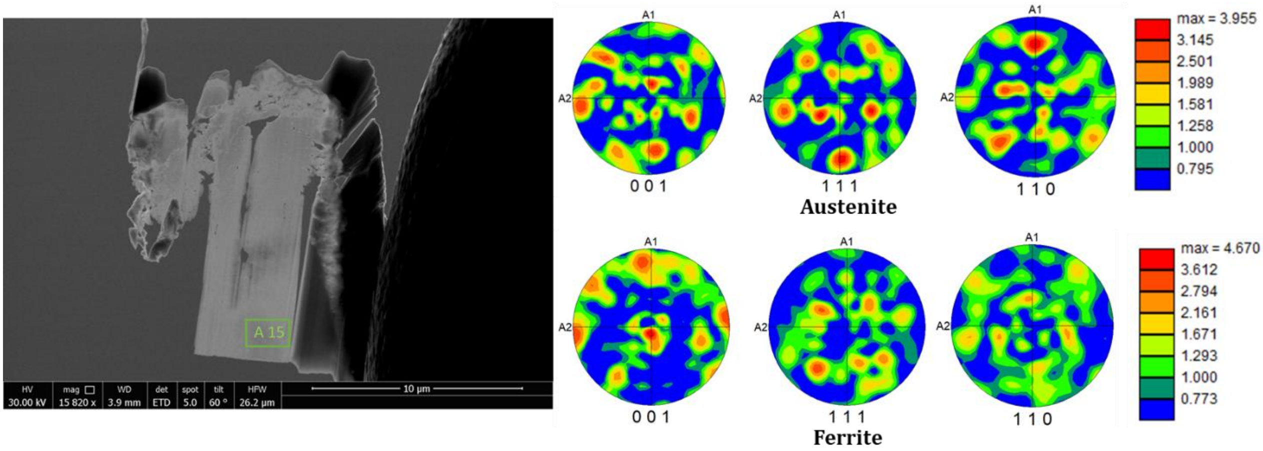

Figure S2. Texture intensity of the samples taken deeper from the laser structured surface (Area of interest marked in green).

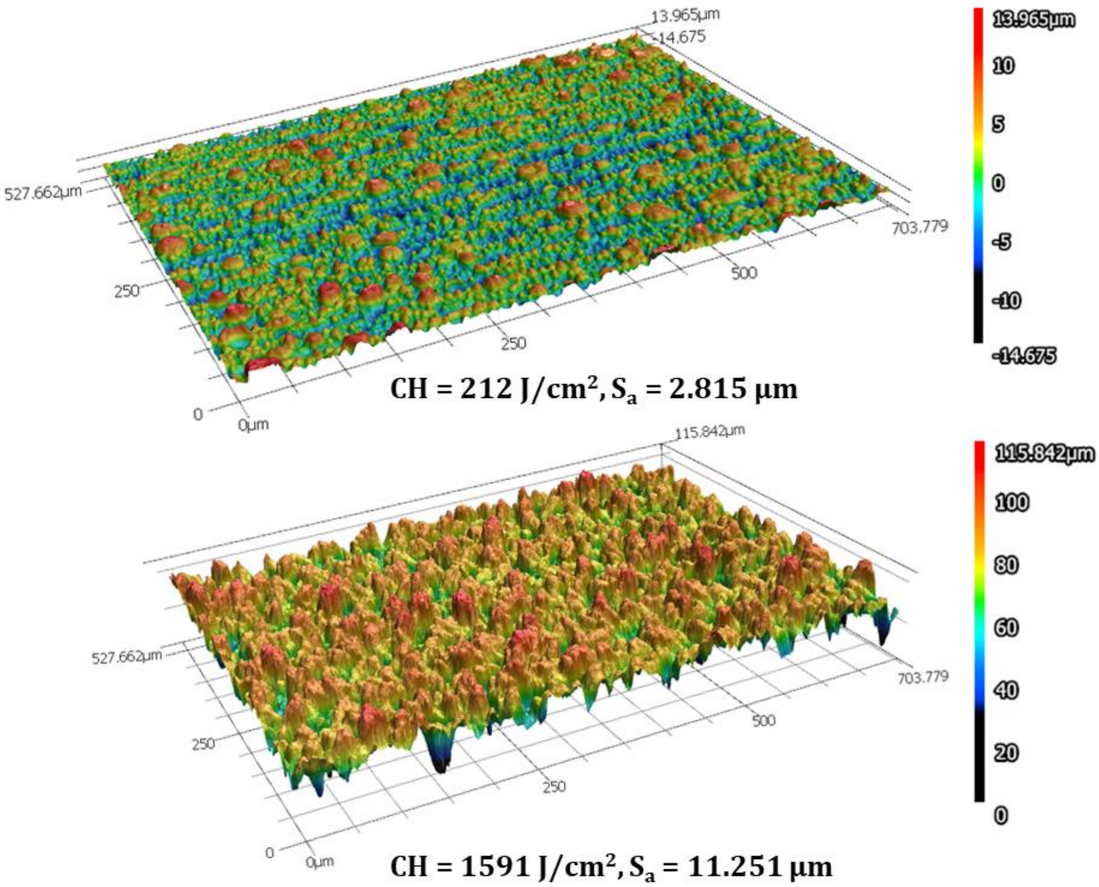

Figure S3. 3D-surface topography and the corresponding S_a values at 212 J/cm^2 and 1591 J/cm^2.
